# Never Say Never: Presumed Inherited Factor VII Deficiency Lies in Wait for 17 Years

**DOI:** 10.7759/cureus.115120

**Published:** 2026-08-24

**Authors:** Austin P Runde, Sofia I Ahmed, Grace C Devitt, Jeremy A Kleberg, Natalie L Kamberos

**Affiliations:** 1 MD Program, Stritch School of Medicine, Loyola University Chicago, Maywood, USA; 2 Internal Medicine-Pediatrics, Loyola University Medical Center, Maywood, USA; 3 Pediatric Oncology, Loyola University Medical Center, Maywood, USA

**Keywords:** extrinsic pathway, factor vii deficiency, fresh frozen plasma (ffp), inherited coagulopathy, pediatric hematology, vitamin k insufficiency

## Abstract

A previously healthy, 17-year-old girl presented with gross hematuria in the setting of a severely deranged coagulation profile: prothrombin time (PT) > 320 s, international normalized ratio (INR) 15, and activated partial thromboplastin time (aPTT) 59 s. Factor VII (F7) activity (FVII:C) was undetectable. Following administration of two units of fresh frozen plasma (FFP) and phytonadione, her FVII:C increased to 38%, and coagulation profile improved to PT 16.0 s, INR 1.4, and aPTT 30.3 s. With the near-total loss of such a critical coagulation factor, we felt it was exceedingly unusual for a patient to present after 17 years without antecedent bleeding. However, testing ruled out inhibitors, which strongly suggested her deficiency was inherited as opposed to acquired. Moreover, we would not anticipate such dramatic rescue of the coagulation profile with FFP if inhibitors were present, even if they were cross-reactive with F7 and another coagulation factor. And with a vitamin K level near the lower limit of normal, we surmised that a vitamin K insufficiency complicated her severe factor deficiency. As such, while uncommon, patients with severe, inherited deficiency of F7 may not experience their index bleed until many years, even decades, after infancy.

## Introduction

Factor VII (F7) is a vitamin K-dependent component of the extrinsic pathway of the coagulation cascade [1, 2]. F7 is activated (F7a) by factor III (tissue factor), the coagulation factor released by endothelial cells following injury. F7a catalyzes the activation of factor X (Stuart-Prower factor) in the common pathway. Like the other coagulation factors, except factors III and VIII, F7 is synthesized by the liver. F7 has the shortest half-life, around 4.5 hours, making it uniquely vulnerable to liver dysfunction and vitamin K deficiency (VKD) [3, 4]. F7 deficiencies (F7D) result in prolongation of the prothrombin time (PT)/international normalized ratio (INR) [2].

Factor VII deficiencies can be inherited (iF7D) or acquired (aF7D). iF7D occurs in an autosomal recessive fashion with an incidence of approximately 1 in 500,000 [4]. In aF7D, deficiencies can either be isolated or non-isolated [5, 6]. Isolated aF7D can occur secondary to the formation of F7-neutralizing autoantibodies (inhibitors). Non-isolated aF7D can occur secondary to reduced synthetic function of the liver (wherein all coagulation factors but factors III and VIII are reduced) or VKD (wherein the vitamin K-dependent factors [II, VII, IX, and X] are selectively reduced).

Only 20% F7 coagulant activity (FVII:C) is required to be phenotypically normal [7]. While more severe iF7D typically leads to earlier presentation, symptomatic patients generally present around 8 years of age [8-10]. Patients with F7D present with menorrhagia, epistaxis, and/or frequent ecchymoses; however, more severe forms may present neonatally or in infancy with hemarthrosis, gastrointestinal bleeding, and/or intracranial hemorrhage [10, 11]. Disease severity correlates with FVII:C: < 10% is severe, 10-20% is moderate, and greater than 20% is mild or asymptomatic. iF7D is often diagnosed after a patient presents with an "index bleed": an unprovoked or otherwise atypical episode of severe bleeding. iF7D may also be diagnosed after patients present with non-life-threatening but recurrent or unusual bleeding, and/or a family history suggestive of coagulopathy [9,10].

Due to symptom overlap with other coagulopathies, iF7D may be clinically similar to other factor deficiencies (*e.g.*, hemophilia A/B/C, hypoprothrombinemia, factor X deficiency) or von Willebrand disease (vWD), as all can present with ecchymoses, hemarthrosis, and epistaxis [12]. Fortunately, laboratory testing is extremely effective at diagnosing coagulopathies.

Workup includes complete blood count (CBC) with differential, PT/INR, activated partial thromboplastin time (aPTT), fibrinogen level, PT/aPTT mixing studies, ristocetin cofactor assay, von Willebrand collagen-binding assay, and potentially even platelet aggregometry. A prolonged PT/INR with normal aPTT is highly suggestive of F7D, as F7 functions exclusively in the extrinsic pathway [9, 12]. To confirm the diagnosis, FVII:C (a one-stage, PT-based clotting time assay) should be performed to measure the level of circulating F7 [9, 10]. iF7D can be further categorized between quantitative/type I or qualitative/type II defects: while both types display decreased FVII:C, F7 antigen level (FVII:Ag; an immunoassay) is decreased in type I but normal in type II.

Factor VII deficiency is treated emergently with fresh frozen plasma (FFP). While FFP is typically successful at normalizing the coagulation profile, the low concentration of F7 in FFP imparts a risk of volume overload when FFP is used exclusively. Moreover, there are inherent immunologic and, to a far lesser degree, infectious risks associated with the transfusion of any blood product. As such, chronic management of F7D with recombinant F7a (rF7a; e.g., NovoSeven^®^, Novo Nordisk, Bagsværd, Denmark) is preferred, though some patients may be managed with prothrombin complex concentrate (PCC) or plasma-derived F7 concentrates [8]. Rarely, inhibitors to rF7a may form, complicating management [12]. While some patients may still be successfully treated with rF7a despite the presence of inhibitors, others may require escalation to activated PCC (e.g., FEIBA^®^, Takeda Pharmaceutical, Tokyo, Japan) if they are unable to respond to rF7a. 

This case report was presented as an abstract at the 2026 American Society of Pediatric Hematology/Oncology (ASPHO) Conference in Minneapolis, MN, USA.

## Case presentation

Our patient was a 17-year-old girl without contributory past medical history. The only surgical/pharmacologic history was an etonogestrel-containing contraceptive implanted subdermally in the proximal left arm 6 weeks prior. She denied prior hospitalizations, allergies, and use of ethanol, tobacco, and recreational/controlled substances.

The patient presented to our pediatric intensive care unit as a transfer from an outside hospital. She sought care initially for sharp, right-sided flank pain, hematuria, and hematochezia of 3 days’ duration. She stated she had also been experiencing a cough, pharyngitis, and pink-tinged phlegm for 4 days prior. She denied recent illnesses, fever, vomiting, joint pain, rhinorrhea, and abdominal pain. She had no recent travel history, sick contacts, history of blood transfusion, or receipt of blood products. She denied personal and family history of hematologic disorders and bleeding diatheses. Her last menstrual period ended 11 days prior. Menstrual periods occurred regularly, lasting 6 days, and required only up to three pads per day; she denied menorrhagia and dysmenorrhea. A review of systems revealed near-daily headaches since implantation of the contraceptive and intermittent, bilateral leg swelling with associated pain in the preceding week, and a history of well-formed, soft albeit floating stools.

**Figure 1:**
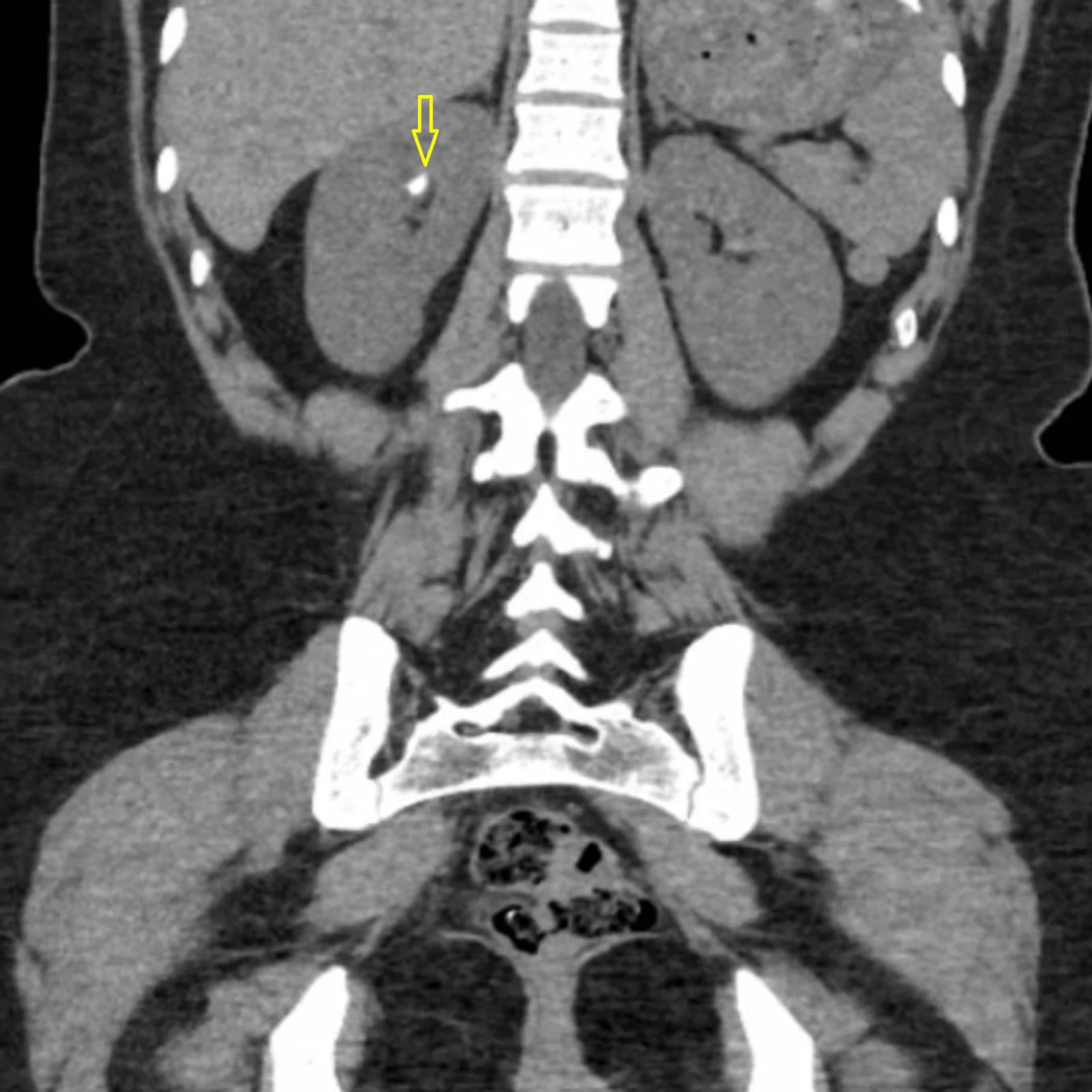
CT scan of the abdomen/pelvis CT scan of the abdomen/pelvis, renal stone protocol (without contrast), coronal view, demonstrating nephrolithiasis of the right kidney (yellow arrow).

**Figure 2:**
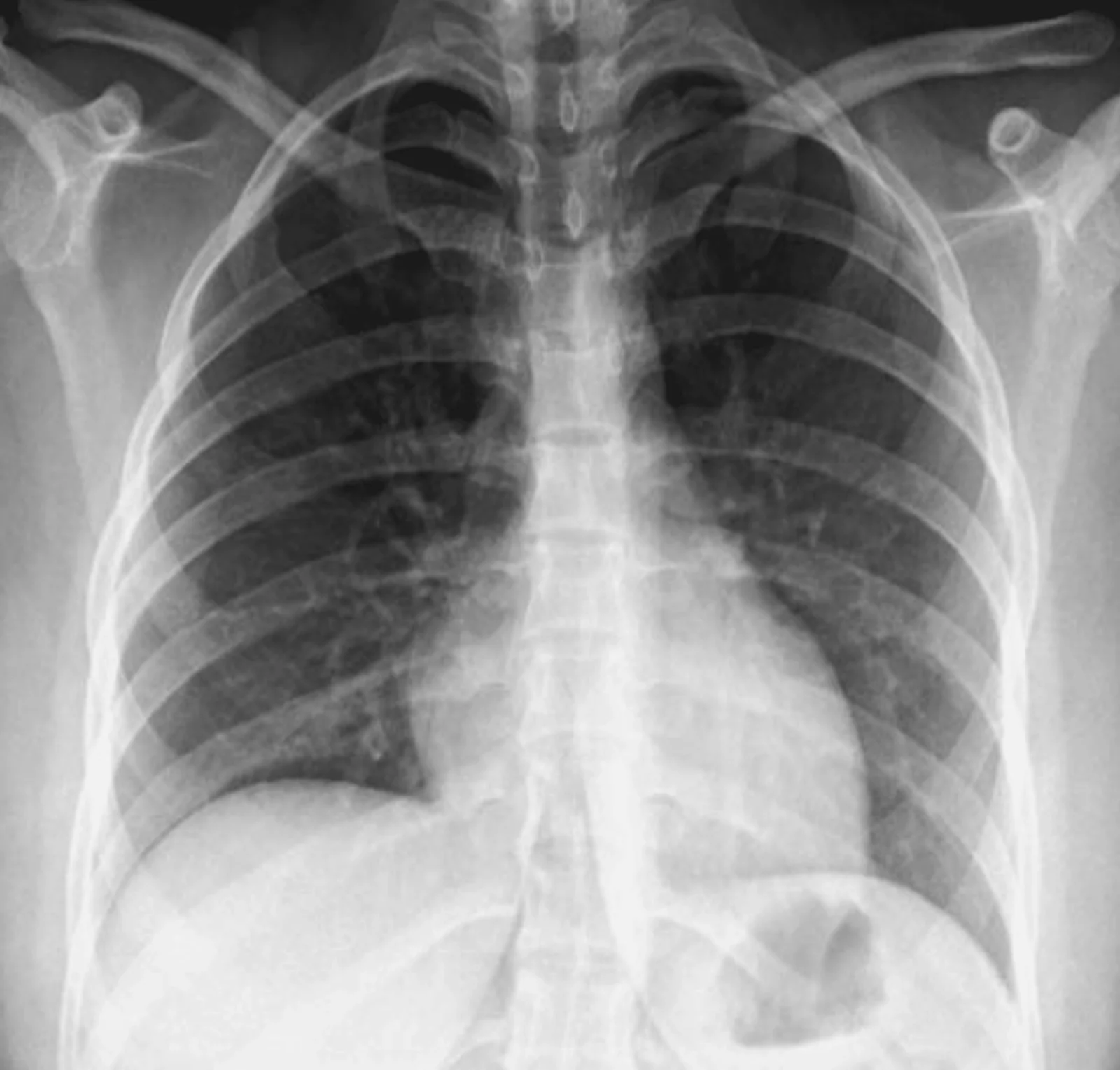
Plain chest radiograph Posterior-anterior, upright, plain radiograph of the chest demonstrating no abnormalities.

She was transferred to our institution when coagulation studies revealed PT > 320 (ref. range: 9.9-12.8 s), INR 15 (ref. range: 0.8- 1.2), and aPTT 59 (ref. range: 24.7-35.7 s) in the setting of urinalysis revealing hematuria > 100 (reference: 0-2 red blood cells (RBCs)/high-powered field (HPF)). Computed tomography (CT) scan of the abdomen/pelvis without contrast revealed an 8-mm nonobstructive stone in the right kidney (Figure 1). Complete blood count (CBC) was remarkable only for hemoglobin 10.6 (ref. range: 11.5-15.5 g/dL). Chest radiograph was unremarkable (Figure 2), and testing for influenza A/B, SARS-CoV-2, and respiratory syncytial virus was negative.

With the derangement of her coagulation profile, the differential diagnosis included: autosomal-inherited factor deficiency (e.g., II, V, VII, X), factor inhibitor, VKD, liver synthetic dysfunction, and lupus anticoagulant (LA). Disseminated intravascular coagulation was considered, though her clinical stability and normal platelets/fibrinogen refuted this diagnosis. With the specific laboratory abnormalities and lack of any history of mucosal bleeding, concern for vWD was minimal.

Vital signs on admission were: blood pressure 136/72 mmHg, heart rate 78 beats/minute, temperature 97.8°F (36.6°C), respiratory rate 16 breaths/minute, peripheral oxygen saturation (SpO_2_) 100% on room air, body surface area (BSA) 1.81 m^2^, and BMI 28.83 kg/m². Physical exam remarkable only for tenderness of the right lower abdominal quadrant, without guarding or rebound, and a 4-cm, healing ecchymosis on the distal left leg.

Abnormal results seen in complete metabolic panel (CMP), CBC with differential, coagulation profile, fibrinogen, FVII:C, serum uric acid, vitamin A/D/K, intact parathyroid hormone, urinalysis with reflexed culture, urine drug screen, pregnancy test, urine calcium, urine creatinine, urine calcium/creatinine ratio, and respiratory pathogen testing are listed in Table 1. Blood type and screen confirmed O(+) and negative alloantibodies.

**Table 1:** Abnormal results from testing performed on admission Hgb, hemoglobin; HCT, hematocrit; MCV, mean corpuscular volume; MCH, mean corpuscular hemoglobin; RDW, red blood cell distribution width; Cr, creatinine; Tbili, total serum bilirubin; FVII:C, factor VII coagulant activity; PT, prothrombin time; INR, international normalized ratio; aPTT, activated partial thromboplastin time; 25(OH)D, 25-hydroxyvitamin D; Ca, calcium; Ag, antigen.

Component	Value	Reference Range and Units
Hgb	10.0	11.5–15.5 g/dL
HCT	31.0	34.0–46.5%
MCV	78.9	82.0–99.0 fL
MCH	25.4	27.0–34.0 pg
RDW	16.1	11.0–15.0%
Serum Cr	0.52	0.6–1.4 mg/dL
Tbili	0.1	0.2–1.4 mg/dL
FVII:C	< 1	> 60%
PT	> 200.0	9.9–12.8 s
INR	> 20.0	0.8–1.2
aPTT	53.8	24.7–35.7 s
Vitamin A	36	38–106 ug/dL
25(OH)D	17	30–80 ng/mL
Serum Ca	8.6	8.0–10.3 mg/dL
*Streptococcus pyogenes* Ag (pharyngeal)	(+)	(-)
*Helicobacter pylori* Ag (stool)	(+)	(-)
Urine clarity	Hazy	Clear
Urine Ca	6.1	0.6-4.0 mg/dL

On admission, two units of FFP were administered, which resulted in partial rescue: PT 42.3, INR 3.8, and aPTT 44.3. Vitamin K (as phytonadione; 10 mg daily, 3-day course (first two doses intravenous, final dose oral)) was initiated empirically for potential VKD considering her coagulopathy in the setting of having endorsed possible steatorrhea. Lactated Ringer’s was initiated at a maintenance rate of 83 cc/hr to maintain hydration in the setting of nephrolithiasis. Amoxicillin (500 mg orally twice daily, 10-day course) was initiated for *Streptococcus pyogenes* pharyngitis. She was incidentally positive for *Helicobacter pylori* (*H. pylori*), which was tested for in the setting of parental concern amid household history; bismuth-based quadruple therapy was planned for initiation in an outpatient setting. Acetaminophen 15 mg/kg every 6 hours, oxycodone 5 mg every 6 hours, and hydromorphone 0.6 mg every 3 hours were available as needed based on increasing pain severity.

Prothrombin time mixing study showed correction after the addition of normal plasma; aPTT mixing study was not deemed indicated by our institution's blood bank. While random urine calcium was elevated, the normal calcium/creatinine ratio strongly refuted the presence of hypercalciuria. Rheumatologic workup was negative for antiphospholipid antibodies, though antinuclear antibody (ANA) was weakly positive at 1:40 (reference: negative) with nuclear envelope-patterned staining. Thyroid-stimulating hormone, iron studies, ferritin, lipase, and B12/folate levels were normal. Importantly, screening for F7 inhibitors was negative.

**Figure 3:**
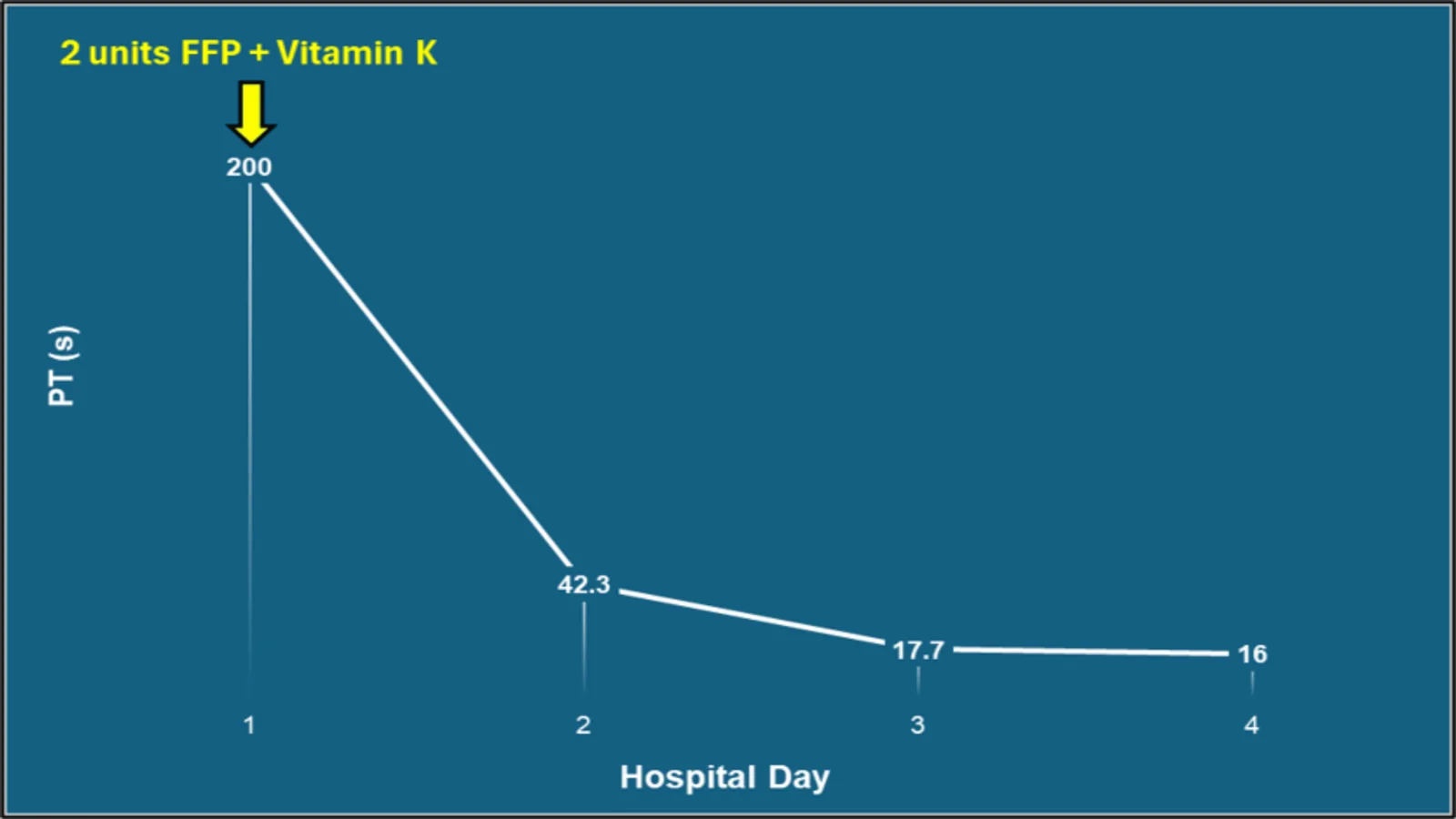
Run chart depicting PT measured daily from admission and intervention (yellow arrow) to discharge. PT, prothrombin time; FFP, fresh frozen plasma

**Figure 4:**
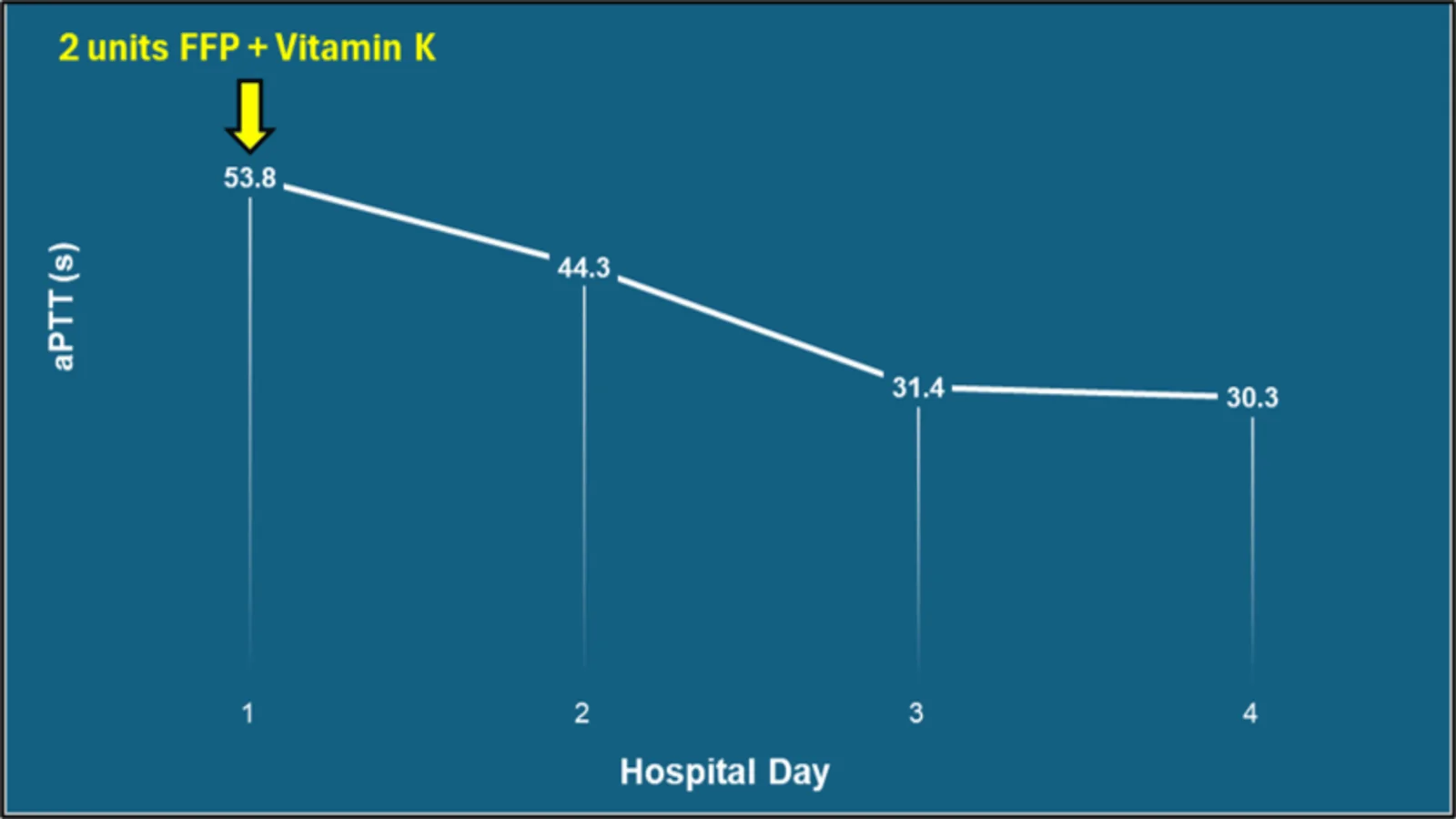
Run chart depicting aPTT measured daily from admission and intervention (yellow arrow) to discharge. aPTT, activated partial thromboplastin time; FFP, fresh frozen plasma

The rF7a was prescribed but initially denied by her insurance; fortunately, emergent use was not necessary with her clinical stability. After a 4-day admission, our patient was discharged home with a resolution of bleeding and significantly improved coagulation profile: PT 16.0 (Figure 3), INR 1.4, aPTT 30.3 (Figure 4), and FVII:C 38%.

She had follow-up appointments scheduled in general pediatrics and pediatric subspecialties. The rF7a was ultimately approved on appeal for medical necessity, and she was scheduled to begin treatment as an outpatient.

## Discussion

We found six cases similar to our patient’s [13-16]. Index bleeds in the reported cases were either unprovoked or provoked by only minor trauma; coagulation profile derangements were found incidentally during pre-surgical testing. Notably, several of the patients who underwent surgery tolerated the procedures without hemorrhage, with FVII:C ranging from 1% to just 9%. And yet, the recommendation for surgery is that FVII:C be at least 15% [13]. Thus, while it is possible for patients with severe iF7D to have their index bleeding event outside infancy, it is exceedingly rare, and the vast majority of iF7D patients with severe deficiency present within the first year of life.

In general, it is not uncommon for patients with iF7D to be mildly symptomatic or even asymptomatic, as there is considerable variability in the severity of iF7D. In fact, an FVII:C just slightly above 20% is required to be asymptomatic [7]. However, our patient presented with bleeding amid undetectable FVII:C. We anticipated it would be virtually impossible for a patient with such severe F7D to have gone 17 years without an index bleed, so we tested for ANA, antiphospholipid antibodies, and F7 inhibitors to rule out aF7D. No inhibitors were detected, and the only laboratory evidence of a rheumatologic process was a weakly elevated ANA (many rheumatologists do not consider an ANA of 1:40 necessitating further evaluation). Moreover, we would not expect such significant rescue of FVII:C after FFP if inhibitors were present. VKD was considered as a potential cause, especially after she endorsed a history of floating stools concerning for fat malabsorption, though her vitamin K and lipase levels were reassuring. Additionally, at least one study has found that *H. pylori* infection is not associated with alterations in blood rheology or with levels of circulating F7, factor VIII, von Willebrand factor, or fibrinogen [17].

It is unusual that our patient also had a prolonged aPTT, as F7D is canonically associated with isolated prolongation of the PT/INR. We do not have a satisfactory explanation to account for this finding. Concomitant prolongation of PT/INR and aPTT typically suggests either antagonism of the common pathway (e.g., factor II/V/X deficiency/inhibitor, VKD, liver synthetic dysfunction) or co-occurring pathologies affecting the extrinsic and intrinsic pathways independently (e.g., iF7D and factor XI deficiency/inhibitor, respectively); although it would be extraordinarily unlikely for a patient to have pathologies of both types, they are not mutually exclusive. In severe cases, vWD can result in prolonged aPTT due to the interaction of von Willebrand factor with factor VIII. Additionally, LA is known to artifactually prolong aPTT and sometimes also PT/INR. The effect of LA on the PT/INR and aPTT was seen in a 36-year-old patient with aF7D who presented with prolongation of both, in contrast to our pediatric patient who also had prolonged PT/INR and aPTT without evidence of LA [18].

Moreover, while our patient’s vitamin K level was technically normal at 0.177 (ref. range: 0.13-1.5 ng/mL), it is marginally above the lower limit of normal. In the setting of an iF7D as severe as hers, we hypothesize her low-normal vitamin K level complicated the coagulopathy, leading to prolongation of the aPTT in addition to PT/INR. Thus, patients with severe iF7D may require serum vitamin K levels above 0.177 ng/mL to prevent decompensation.

It is also possible there is a cross-reactive inhibitor neutralizing both F7 and a factor of the intrinsic and/or common pathway(s), though we would expect our F7 inhibitor screen to reveal any such autoantibody; and again, if this were an inhibitor-mediated coagulopathy, we would not expect such significant rescue with FFP and phytonadione.

Given our patient's presentation and laboratory findings, we suspect she has a severe iF7D. However, given the prolongation of both PT/INR and aPTT, we acknowledge that the inherited nature of her F7D is presumed. The confirmation for iF7D in this case would be genetic testing of the *F7* gene. However, considering the odds of having both iF7D and another coagulopathy, such as vWD, it is far more likely our patient has iF7D, complicated by insufficient vitamin K, and simply did not experience an index bleed until now. Alternatively, we acknowledge the theoretical existence of an inhibitor directed against both F7 and a component of the common and/or intrinsic pathway(s); however, the significant rescue seen with FFP would be unlikely if such an inhibitor(s) were present. Regardless, we acknowledge these as limitations of our case study but still presume this to be iF7D.

## Conclusions

As such, our patient's case demonstrates that even those with a near-total deficiency of F7 may not present until nearly their second decade of life or later. How this is possible, we are unsure. This is an excellent illustration of the complexity of biology.

We report this case to reinforce the necessity of clinicians being open-minded and not ruling out inherited disorders based on the patient’s age at index presentation. We also present evidence, albeit anecdotal, to suggest patients with severe iF7D may have a relatively increased basal requirement for vitamin K. Additionally, we provide a striking example of a patient with a severely deranged coagulation profile who presented as hemodynamically stable with relatively mild symptoms.
